# Synthesis and Characterization of Soy Hull Biochar-Based Flexible Polyurethane Foam Composites

**DOI:** 10.3390/ma18092006

**Published:** 2025-04-29

**Authors:** Kavya Ganesan, Bethany Guin, Elijah Wilbanks, James Sternberg

**Affiliations:** Department of Food, Nutrition, and Packaging Science, Clemson University, Clemson, SC 29634, USA; kavyag@g.clemson.edu (K.G.);

**Keywords:** biochar, flexible polyurethane foams, flammability, sustainability

## Abstract

Flexible polyurethane foams are a diverse class of materials encompassing furniture, packaging, automotive, and many other industrial and domestic applications. Polyurethane foams are synthesized by the addition of polyols and isocyanates; however, the petroleum origin and toxic nature of isocyanates have driven many to look for more sustainable routes to production. Renewable fillers have emerged as a biobased resource to decrease the carbon footprint of this widely used polymeric material. In this study, soy hulls, as mass-produced, industrial by-products of soybean production, were used to create a biochar beneficial in the synthesis of flexible polyurethane foam composites. The addition of soy hull biochar was found to maintain the compression properties of foams at a decreasing isocyanate index, reducing the amount of isocyanates needed for production. In addition, the addition of biochar decreased the flammability of foams, important for many applications where consumer safety is important. The results point to the ability to create safer, more sustainable, and even more cost-effective polyurethane foams through the reduction in isocyanate use while maintaining the properties of this important class of polymers.

## 1. Introduction

Polyurethane (PU) foams are a class of versatile materials made from isocyanates and polyols that can be varied to achieve properties from rigid to flexible and elastomeric. PU foams are used extensively in the automotive seating, furniture, appliance, construction, and apparel markets due to highly developed cellular networks that impart impressive physical, mechanical, and insulative properties for diverse commercial applications. Flexible foam represents 31% of the PU foam market and is primarily used in comfort applications for seating, bedding, and other cushioning applications [1].

PU foams are primarily synthesized from petroleum-based chemicals with a significant carbon footprint. Given the dangers of global warming and increased desires to move to carbon neutrality, many researchers and commercial suppliers are looking to renewable sources for polyurethane production [2]. Polyurethanes are typically synthesized from a two components system made up of a “Part A” mixture of polyols, surfactants, catalysts, and blowing agents to a “Part B” isocyanate component that initiates the reaction [1]. While rigid foams make use of shorter polyols with a higher hydroxyl content, flexible foams are composed of longer aliphatic precursors with hydroxyl values typically below 100 mgKOH/g [3]. The combination of surfactant, polyol structure, and catalyst amount is able to create materials with densities below 30 kg/m^3^ for lightweight applications or introduce viscoelastic properties for shape memory and cushioning characteristics [4].

The first biobased foams were initially produced by functionalizing vegetable oils with hydroxyl groups to react with isocyanates [5]. Foams with similar properties to non-biobased materials were created up to around 50% polyol replacement of the vegetable oil-based precursor [6]. Since then, examples of biobased PU foams can be found based on carbohydrates and cellulose, lignin and tannins, glycerol from biorefining, and other sources [3]. Biobased PUs can result from the chemical modification of biobased sources to participate in the reaction toward the urethane polymer network or exist as fillers that can bolster the mechanical properties of PU foams and act as nucleating agents during the nascent development of the cellular morphology [7].

In recent years, considerable research has focused on the incorporation of bio-based fillers to enhance the sustainability and functionality of PU foams. Fillers such as nanocellulose [8], lignin [9], hemp fibers [10], starch [11], and agricultural residues [12] have been explored for their potential to modify mechanical strength, thermal stability, biodegradability, and flammability properties of both rigid and flexible PU foams. For instance, Recupido et al. [10] demonstrated that rigid PU foams reinforced with functionalized hemp particles exhibited enhanced compressive strength, while those incorporating phytic-acid-modified silica showed significant improvement in fire resistance and thermal stability. However, many such studies are either focused on rigid foams or specific filler types and rarely address comprehensive structure–property relationships or the effect on flexible compression properties.

Soy hull-derived biochar, in contrast, represents a unique filler material due to its low cost, carbon-rich and porous structure, and renewable agricultural origin. Compared to other biomass-based fillers, biochar typically requires minimal post-processing, while naturally offering surface functionality conducive to reinforcing polymer matrices.

Biochar is a porous carbon-rich material produced through pyrolysis or gasification of biomass, typically at temperatures between 400 and 600 °C [13]. It has attracted increasing interest for its multifunctional properties and environmental benefits. Biochar has demonstrated effectiveness as an adsorbent for toxins in wastewater, a catalyst in air scrubbing systems, and a soil amendment in agricultural systems [13]. Recently, it has also gained traction as a sustainable, low-cost filler in polyurethane (PU) foams for environmental remediation and material performance applications. Numerous studies have highlighted the adsorption performance of biochar-based PU foams for the removal of toxins [14,15], solvents [16], oils [17,18], and dyes [19,20] from aqueous systems. Moreover, these foams show potential in acoustic insulation due to their porous architecture [21].

From a life cycle and sustainability perspective, biochar offers several advantages over conventional additives. According to Bergman et al., biochar systems can result in net environmental benefits when sustainably sourced and efficiently produced, particularly by diverting forest residues from open burning or decomposition, which would otherwise emit large amounts of greenhouse gases (GHGs). Bergamand et al. also emphasize the importance of considering the full life cycle of biochar—feedstock acquisition, pyrolysis, transport, and end-use—to accurately assess its environmental impacts. Biochar can contribute to carbon neutrality or even net-negative emissions through long-term carbon sequestration, as well as the displacement of fossil-fuel-derived materials and energy [22]. These findings support earlier assessments by Gaunt et al., who outlined that beyond direct soil carbon sequestration, additional GHG reductions may result from avoided emissions, enhanced crop efficiency, and fossil fuel displacement [23].

Economically, biochar is an appealing alternative due to the abundance of low-cost biomass feedstocks and its relatively low processing requirements, making it an accessible option for value-added applications in industry. Furthermore, the addition of biochar to PUs can be accomplished without the addition of solvents, compatibilizers, or other costly additives. However, the addition of filler material to PU formulations does change the viscosity and reactivity of the curing system, requiring studies to discover the structure–property relationships between biochar addition and the resulting foam performance.

Despite its growing application potential, the structure–property relationships of biochar–PU foam composites are not yet well understood. One study on rigid PU foams found that incorporating up to 20% biochar reduced compressive strength but maintained thermal insulation properties compared to the neat foam [24]. However, research on flexible PU foams remains missing, underscoring the need for deeper exploration into how biochar influences foam morphology, mechanical resilience, and performance in diverse application settings.

In this study, biochar derived from soy hulls was utilized as a filler material in flexible PU foam formulations. Soy hulls, an agricultural by-product accounting for approximately 5 wt.% of soybeans, are produced at a volume of 17 million tons annually [6]. Traditionally, soy hulls are used as a low-value filler in animal feed, serving as a “soft” lignocellulose biomass source. In this work, biochar was incorporated in flexible PU foams up to 20 wt.% and the physical, mechanical, and thermal properties were tied to morphological characteristics observed in SEM. In addition, this study explores the ability to lower the isocyanate ratio of biochar PU foams while maintaining the essential compression properties of the materials to create a less toxic and more cost competitive composite.

## 2. Materials and Methods

### 2.1. Materials

Soy hulls were obtained from Archer Daniels Midland Co. (Chicago, IL, USA) and were used as received without washing or pretreatment. The polyol blend (OH value 163.2 mg KOH/g) and isocyanate, polymeric methylene diphenyl diisocyanate, (29.8% NCO), were kindly provided by Huntsman (The Woodlands, TX, USA).

### 2.2. Synthesis and Characterization of Soyhull Biochar

The hulls were carbonized in 1 kg batches in a nitrogen atmosphere using a stainless- steel container inserted inside an indirect-fired rotary furnace. The furnace was first heated to 500 °C before the stainless-steel container filled with soy hulls was inserted into the retort of the furnace. This method was used after monitoring the temperature inside the stainless-steel container, and we found that the interior of the container reached the carbonization temperature after approximately 40 min, referring to an approximate 10 °C/min heating rate. The soy hulls were allowed to carbonize at 500 °C for 60 min; then, the insert was removed and allowed to cool down under nitrogen flow. The biochar was then dispensed in a stainless-steel bucket, flushed with nitrogen, and reserved for further use. Biochar was produced from 1 kg of soy hulls and yielded a predictable amount between 25 and 28%.

### 2.3. Preparation of PU Foams

PU foams were prepared using the one-shot method, where the polyol blend and isocyanate were mixed directly in an open cup and allowed to rise and cure at room temperature. Composites were produced by manually grinding the biochar with mortar and pestle and sifting through a 100-micron sieve. Biochar was then hand-mixed with the polyol component at room temperature. Biochar was added at 0, 1, 5, 10, and 20 wt.% of the total weight of polyol and isocyanate combined. Initial foams were produced using a 100% isocyanate index based on the hydroxyl value of the polyol and isocyanate content of pMDI. For the study based on reducing the amount of isocyanate, the isocyanate index was reduced by 11.1% each trial for a series of foams ending with an isocyanate index of 33.

### 2.4. Characterization

Foam density was calculated using 3 measurements of the dimensions of each sample. Compression testing was performed on 4 foams with varying biochar and isocyanate contents using the compressive force deflection method found in ASTM D3574-17. The dimensions of the foam were approximately 25 mm × 25 mm × 25 mm. Infrared spectra were collected on Thermo Nicolet iS10 (Waltham, MA, USA) from 500 to 4000 cm^−1^ using 16 scans with a spectral resolution of 4 cm^−1^. Differential Scanning Calorimetry (DSC) was completed on a TA Instruments DSC250 to evaluate the Tg of the samples by estimating the midpoint of the slope change in the heat flow vs. temperature curve. A heat–cool–heat curve with a heating rate of 10 °C from 20 °C to 200 °C was completed. Thermogravimetric Analysis (TGA) was completed on a TA Instruments Q5000 (New Castle, DE, USA) at a heating rate of 10 °C/min from 25 to 700 °C under a nitrogen atmosphere. Scanning Electron Microscopy (SEM) analysis was performed on Hitachi 3400 (Tokyo, Japan). Samples were sputter coated with platinum prior to imaging and subsequently imaged at an acceleration voltage of 10 kV. Burn testing was conducted according to ASTM D4986-22 with modification to the foam size. Samples were cut to approximately 25 × 22 × 13 mm (l × w × h) and ignited using a wing-tipped flame using natural gas. The time taken to burn to the 4 mm, 10 mm, 21 mm, and entire length of sample was noted along with the burn time after the flame was removed and if the cotton placed below the sample was ignited by burning residue.

## 3. Results and Discussion

**Figure 1:**
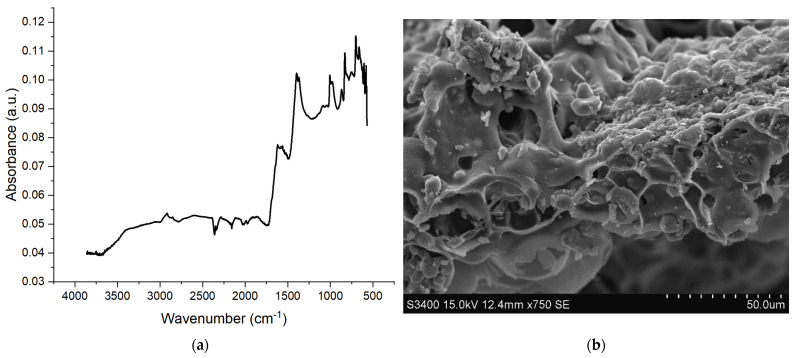
(**a**) FTIR spectra and (**b**) SEM image of biochar used.

Soy hulls were successfully carbonized using a simple pyrolysis procedure at 500 °C with an approximately 25% yield. The biochar exhibited relatively low porosity and high ash content (Table 1) stemming from its agricultural source. CHNS analysis revealed the biochar contained 55.16% carbon but a high ash content of 16.6%. It can be assumed that the remaining oxygen content was around 23%, pointing to significant functional group content in the biochar. The biochar was easily ground and sieved to a particle size of less than 100 microns and mixed well with the polyol used in this study. The SEM images (Figure 1b) of the biochar reveal a highly porous and irregular surface morphology, characteristic of pyrolyzed biomass, which enhances surface area and suggests potential for adsorption or reinforcement applications. Fourier-Transform Infrared (FTIR, Figure 1a)) spectroscopy confirms the presence of functional groups on the biochar surface. A broad peak around 3400 cm⁻^1^ corresponds to –OH stretching from hydroxyl groups. The region between 1500 and 1700 cm⁻^1^ shows strong absorption due to aromatic C=C stretching and carbonyl (C=O) vibrations. Notable peaks between 1200 and 1400 cm⁻^1^ suggest C–H bending and C–O stretching from phenolics or aliphatic structures These peaks indicate the presence of conjugated aromatic structures and oxidized functionalities. Such groups originate from the degradation of lignin and carbohydrates during pyrolysis. These functionalities enhance the surface reactivity and chemical compatibility of the biochar. Together, SEM and FTIR analyses provide insight into the porosity and surface chemistry of the biochar revealing a filler material apt for use in composite applications.

Two separate studies were conducted to understand the effect of biochar addition to a flexible PU formulation. In the first study, biochar was added to a PU formulation with stoichiometric equivalence of polyol and isocyanate (Iso index of 100) to determine the changes in the thermal and mechanical properties by increasing biochar content to 20%.

After an optimal biochar percentage was determined, a series of foams were synthesized with decreasing isocyanate content to assess if biochar could enable the reduction in isocyanate needed in flexible PU foam formulations. Burn testing of foams was also completed to assess the flame retardant properties of the biochar, important for commercial PU applications.

### 3.1. Biochar Composites at 100 Isocyanate Index

The density and compression strength of flexible PU foams are important with regard to their lightweight status and cushioning/impact resistance when used in packaging and seating applications. Compression strength typically follows a linear relationship with density until the densification region where foam cells have been compressed completely and compression begins to be applied to the cell walls themselves [18]. In this study, compression strength was taken at 50% compression, and compression force deflection was taken after holding at 50% compression for 60 s. The difference between compression strength and compression force deflection reflects the compressive stress relaxation after 60 s. The addition of biochar had very little effect on density and compression strength up to 5% concentration (Table 2). At 10% concentration, a modest increase in density and CFD is observed, followed by a significantly higher increase at 20% concentration, whereas at 10% concentration, the CFD value increases by about 30% from control foams with 0% biochar; at 20% biochar, the value is nearly three times as high as the control. The enhanced compressive strength observed at 20% biochar loading can be attributed to a combination of factors. The significant increase in foam density, from ~60 to ~77 kg/m^3^, plays a key role, as compressive strength is closely linked to density. Additionally, at higher loadings, biochar likely forms a more continuous filler network that reinforces the polymer matrix, improving load transfer and stiffness. Well-dispersed biochar particles may also contribute to stronger matrix–filler interactions, further enhancing mechanical performance. Despite some disruption to cell morphology, the rigid biochar particles may strengthen the foam’s struts and walls, offsetting structural irregularities. This aligns partially with Uram et al. (2021), who reported improved thermal and dimensional stability with biochar but noted reduced strength due to lower density and thinner cell walls [24]. The findings here suggest that, in flexible foams, the reinforcing effect of biochar can dominate over morphological disruptions at higher loadings, resulting in significantly improved compressive performance.

**Figure 2:**
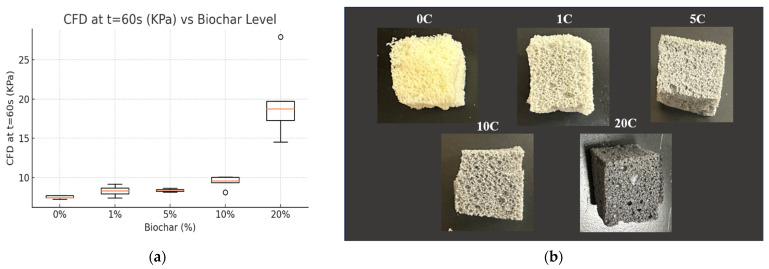
(**a**) One-way ANOVA boxplot for CFD at different biochar concentrations. (**b**) Representation of composite foams with biochar content from 0 to 20% at the 100 Iso Index.

**Figure 3:**
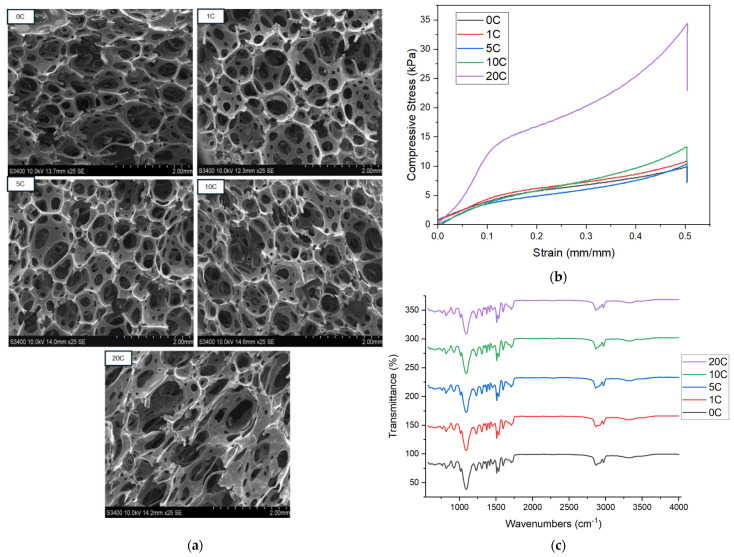
Increasing biochar content from 0 to 20 wt.% (0 C–20 C). (**a**) SEM image of foams; (**b**) CFD of foams; (**c**) FTIR of foams.

Biochar changes the color of foams with increasing concentration (Figure 2b), and small particles can be observed in the cell walls of SEM images reflecting biochar addition (Figure 3a). In all biochar concentrations, the open-cell nature of the foam is maintained with a slight increase in average cell size up to 10%. At 20% biochar, a larger increase is observed in cell size, as well as a more irregular cell morphology, reflecting some disruption to the foaming process. This aligns with the findings of Uram et al., who reported that increasing biochar content influences the initial viscosity and reduces the reactivity of the polyurethane system, leading to modified foam formation and smaller cell cross-sections. Despite these morphological changes and reduced compressive strength at 10% strain, Uram et al. found that biochar-enhanced foams retained good thermal conductivity and showed improved thermal and dimensional stability, supporting their potential use as insulation materials and structural fillers where heat transfer control is needed [24].

The addition of biochar initially decreases the amount of stress relaxation over 60 s (comparing C.S. at t = 0 and CFD at t = 60 s, Table 2) as result of reinforcing the polymer matrix in the cell walls of the foam. However, at higher concentrations (10–20% biochar) larger stress relaxation is observed, pointing to the disruption of natural polymer interactions and entanglement. A one-way ANOVA of the CFD values for each biochar concentration was conducted to create a box plot representing the 25th and 75th percentile of the data, along with the mean (represented by a red line) and outliers (Figure 1a). It is clear from Figure 1a that CFD results up to 10% biochar concentration are very similar, showing minor increases in stiffness to the foams. However, at 20% concentration, even the outlying values for CFD are well above all other results. The large increase in stiffness at 20% biochar value can be observed visually through the CFD plots in Figure 3b. Flexible foams typically observe a linear viscoelastic compression region followed by a plateau region and, finally, a densification region where an exponential rise in compression force is observed [25]. Figure 3b shows that typical compression behavior is maintained at all concentrations; however, at 20% biochar the densification region becomes more prominent as the biochar creates less free space within the foam and reaches the densification region at lower compressive strain. Initially, it can be observed that at 10% biochar addition, the density and compression properties of the flexible foams are largely maintained, indicating an acceptable concentration of biochar without major changes to the desired properties.

FTIR (Figure 3c) displays the main peaks associated with PUs, including the urethane signal around 1725 cm^−1^, C-O stretching at 1100 cm^−1^, and the methylene signals at 2950 cm^−1^. It is important to observe no major changes in the FTIR spectra with increased biochar content, verifying that the curing reaction between isocyanate and polyol is not disrupted. Particularly, the residual isocyanate signal remains absent at 2250 cm^−1^ in all formulations, indicating no major disruption to the polymer formation with increasing biochar content.

**Figure 4:**
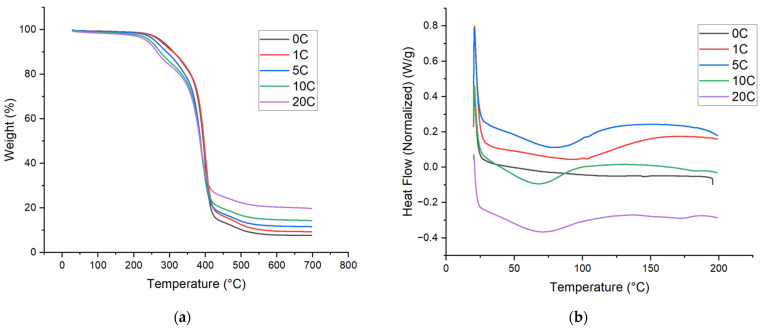
(**a**) TGA of foams; (**b**) DSC of foams from 0 to 20 wt.% biochar (0–20 C) at the 100 Iso Index.

The thermal properties of the PU foams were seen to decrease upon addition of the biochar. DSC scans (Figure 4b) showed no melting endotherms but did witness a decreasing glass transition temperature with biochar increase. A decrease in T_g_ can be explained by the filler’s ability to disrupt polymer interactions in the matrix, causing increased molecular movement. Notably, the glass transition temperature remains above ambient conditions expanding the usable temperature range of application. TGA (Figure 4a) revealed a 2-step degradation pathway typical of PUs where the initial weight loss is associated with dissociation of urethane bonds and volatilization of the polyol component, followed by the breakdown of the isocyanate component. The polymeric methylene diphenyl diisocyanate used in this study is an aromatic precursor with higher heat stability than the aliphatic nature of the polyol. The 5% weight loss (T_d5%_) of the foams decreases with the addition of biochar (Table 3). However, the decrease from 277.9 °C to 235 °C would most likely not have any effect on the use of the foams as the application range of flexible foams is typically in ambient conditions, not in high-heat environments.

Burn testing showed an overall decrease in the burn rate with increasing biochar content (Table 4). It is well known that carbonaceous products can form an intumescent layer during the combustion of polymer materials, reducing the availability of oxygen and substrate available to burn [19]. According to Das et al., the addition of biochar reduces the flammability of polyurethane foams by enhancing thermal stability and promoting char formation [26]. Biochar acts as a heat-resistant barrier, slowing down thermal degradation and forming a protective char layer that insulates the foam and limits oxygen penetration. Its high carbon content and porous structure improve flame resistance by reducing the release of flammable gases and delaying combustion [27]. The burn rate at 10% biochar is approximately 62% that of the control foam with 0% carbon, demonstrating the flame-retardant properties of the biochar. Afterburn time reflects how long the foam composition sustains combustion after the flame is released from the material. While shorter times typically reflect less flammable materials, a longer burn time may also reflect a material that supports combustion at a slower rate. The afterburn time of the foams does increase at higher biochar concentrations (10%, 20%); however, the observation of charred remains after the test vs. the completely combusted material at lower biochar concentrations signifies a slowly burning material after flame removal that eventually extinguishes itself without undergoing complete combustion. Cotton ignition showed no discernable pattern with the control and all biochar concentrations, having at least one sample igniting cotton, aside from the 5% biochar concentration. Overall, biochar significantly reduced the flammability of the foams by decreasing the burn rate and creating materials with self-extinguishing properties, desired in consumer applications. 

The addition of biochar is meant to increase the sustainability of flexible PU foams by increasing biocontent while maintaining the essential properties of the incumbent material. In flexible foams, increasing strength is not generally desired as the cushion properties are associated with flexibility. Here, we have demonstrated that biochar can be added up to 10% concentration while maintaining the compression properties and density of materials, while decreasing the flammability. While other thermal properties such as T_g_ and T_d5%_ do decrease, they remain in usable ranges for flexible foam applications. In the next section, we will explore if the addition of biochar can allow less isocyanate to be used in foam formulations while maintaining their essential properties. As isocyanates are the most expensive component in polyurethane synthesis, decreasing their needed amount would create lower cost materials.

### 3.2. Decreasing Isocyanate Index

The isocyanate portion of a PU foam represents the reactive component of its formulation. Isocyanates and polyols react spontaneously to release heat and increase the rate of the curing reaction that increases entanglement and crosslinking up to the gel point where solid-like properties of the reaction mixture exist [3]. Furthermore, the reaction of isocyanates and water during the foaming reaction releases even more heat than the curing reaction [3], further increasing the kinetics of the growing foam. Decreasing the amount of isocyanate in a PU foam formulation has the potential to decrease crosslinking and entanglement, as well as releasing less gas during the foaming reaction, creating materials with higher density and weaker mechanical properties. Biochar has the potential to mitigate these effects by reinforcing the polymer matrix while also acting as a nucleating agent to encourage gas evolution.

**Figure 5:**
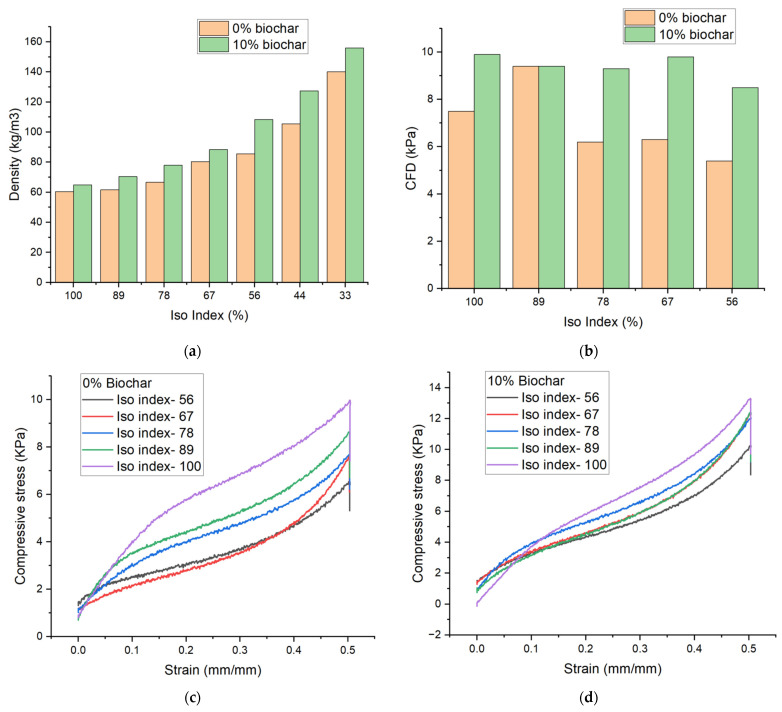
(**a**) Density for decreasing Iso Index for foams with 0% and 10% biochar; (**b**) CFD for decreasing Iso Index with 0% and 10% biochar; (**c**) CFD curve for foam with decreasing Iso Index and 0% biochar content; (**d**) CFD curve for foam with decreasing Iso Index and 10% biochar content.

Foams were created with a decreasing isocyanate index (Iso Index, decreasing by approximately 11.1% each trial) at the 10% biochar concentration due to the favorable properties demonstrated in the initial studies. The biochar composites at a lower Iso Index were compared to foams created at the same index without biochar. Figure 5a shows that a steady increase in density was observed for both control foams with 0% biochar and composites with 10% biochar. A rather larger increase in density (Figure 5a) is observed after an Iso Index of 67, raising the 10% biochar foams above 100 kg/m^3^, possibly demonstrating a limit to the amount of isocyanate that can be decreased. Figure 5b demonstrates the change in CFD value with a decreasing Iso Index, stopping at an index of 56 due to the inability to conduct meaningful CFD analysis due to the decreased structural integrity of foams at an index of 44 and 33. While the control foams without biochar undergo a significant decrease in CFD value, the foams with 10% biochar largely maintain their compressive properties. Similar to the trend with density, after an Iso Index of 67, a decrease in CFD value is observed in composite foams, even though density increased, pointing to a breakdown in the polymer and foam structure leading to diminished physical and mechanical properties. CFD curves (Figure 5c,d) demonstrate the close alignment of composite foams with the decreasing isocyanate index while foams without biochar demonstrate losses in CFD value for each decrease in the isocyanate index. Overall, while an increase in density is observed with biochar addition, up to 33% less isocyanate (Iso Index of 67) is able to be used while maintaining compression properties. This suggests that soy hull biochar acts as a reinforcing filler, compensating for the reduced crosslinking and polymer network density that typically result from lower isocyanate levels. Specifically, the rigid structure of biochar particles likely contributes to cell wall reinforcement and improved stress distribution within the foam. These particles may also facilitate physical entanglements with the polymer matrix, partially offsetting the loss of chemical bonding due to reduced isocyanate. Furthermore, biochar’s high surface area could promote better filler–matrix interactions, supporting the mechanical framework of the foam. This effect is particularly evident at Iso Index values down to 67, where up to 33% less isocyanate is used without significant compromise in compressive properties. Even at an index of 56, some compressive integrity is retained, demonstrating biochar’s potential to extend the formulation window of flexible PU foams while lowering environmental impact and production costs.

Of course, further testing based on compression set and stress relaxation may be needed to validate composites targeted for specific applications; however, these results demonstrate that biochar addition to flexible PU foams are able to largely maintain their physical and mechanical properties with a decreasing Iso Index. Decreasing the amount of isocyanate needed to create foams with similar properties would relate to an overall lower carbon footprint by decreasing the amount of petroleum-derived monomers needed for production. A decrease in isocyanate would also relate to a lower cost in production, as the isocyanate portion is typically the most expensive component to PU production.

**Figure 6:**
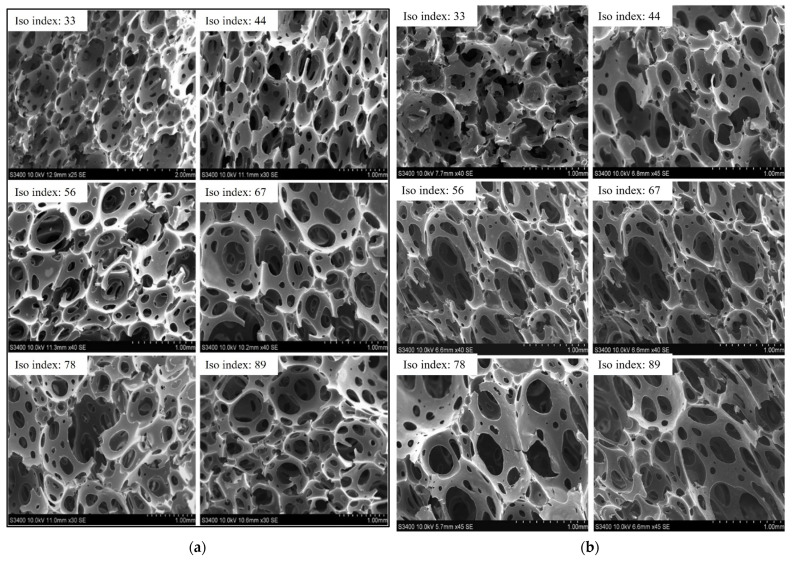
SEM images of foam cells for varying isocyanate (**a**) 0% biochar; (**b**) 10% biochar.

Analysis of the composite foam structures at a decreasing isocyanate index using SEM (Figure 6a,b) demonstrated that the cellular morphology is largely maintained until an Iso Index of 56, after which a significant increase in cellular rupture and disconnected cells can be observed. As with CFD results, the SEM images confirm that beyond an Iso Index of 56, significant deviations occur, including disruption in cellular morphology and structural integrity of the polymer matrix in the struts of cells. The increase in density observed at a lower Iso Index (Figure 5a) is related to the collapse of the normal cellular structure observed at a higher Iso Index, where cellular collapse leads to less free space in the foams and more dense materials.

Burn testing results at a lower Iso Index (Table 5) do not show any major changes to burn rate from foams synthesized at the 100 index. The burn rate is maintained around 1 mm/s for this series compared to 0.76 mm/s for foams synthesized at the 100 index. Foams with a decreasing Iso Index were consumed completely and did not demonstrate the same self-extinguishing properties as those at the 100 Iso Index. This could be due to the decrease in structural integrity witnessed in SEM images, as the ability to form an intumescent layer during combustion is partially dependent upon the integrity of the combusting material. Thus, material with less structural integrity would collapse or form pores easier, supporting continued combustion.

## 4. Conclusions

This study successfully demonstrated the incorporation of soy hull-derived biochar as a sustainable filler material in flexible PU foams, offering an innovative use for an abundant agricultural by-product. Soy hull biochar at 10% concentration was able to reinforce the polymer matrix of PU foams without significantly changing their flexible properties. The addition of biochar decreased the flammability of foams and allowed for 33–44% less isocyanate to be used in formulations with similar physical and mechanical properties. While further mechanical testing would be required to validate biochar PU composites for specific applications, this study demonstrates that biochar is compatible with flexible foam formulations and can be used to increase bio-content and decrease the use of isocyanates, potentially creating more cost-competitive and eco-friendly foam products. From a life cycle and environmental impact perspective, biochar offers several key benefits. When produced sustainably—such as from agricultural or forest residues—it can reduce greenhouse gas emissions by diverting biomass from open burning or decomposition. Biochar also contributes to long-term carbon sequestration, potentially enabling carbon-neutral or even net-negative emissions. Additionally, its use can displace fossil-fuel-derived materials, further lowering the environmental footprint. These advantages make biochar a promising additive for sustainable material development, provided its full life cycle—from feedstock sourcing to end-use—is carefully managed.

## Figures and Tables

**Table 1:** BET surface area, pore distribution, and ash content of soy hull biochar.

**Sample**	**BET Surface Area (m^2^/g)**		**Total Pore Volume (cm^3^/g)**		**Ash Content (%)**
Soy Hull Biochar	5.16 ± 0.56		0.0125 ± 0.0007		16.6 ± 0.36
	Nitrogen (%)	Carbon (%)		Hydrogen (%)	Sulfur (%)
	1.84 ± 0.06	55.16 ± 0.28		3.06 ± 0.05	0.00

**Table 2:** Density, cell length, compression strength (C.S.), and CFD for varying biochar content at Iso Index 100.

Biochar (%)	Density(kg/m^3^)	Cells Length (mm)	C.S. at t = 0 s (KPa)	CFD at t = 60 s (KPa)	Compressive Stress Relaxation (%)
0	60.35 ± 4.94	0.83 ± 0.09	9.87 ± 0.21	7.52 ± 0.2	23.8
1	61.12 ± 3.64	0.91 ± 0.10	11.04 ± 1.41	8.48 ± 0.96	23.1
5	61.63 ± 2.23	0.93 ± 0.22	10.81 ± 0.43	8.35 ± 0.14	22.8
10	64.92 ± 4.18	0.85 ± 0.08	13.81 ± 1.58	9.92 ± 0.94	28.1
20	76.99 ± 13.53	1.18 ± 0.31	32.47 ± 6.24	21.65 ± 4.25	33.3

**Table 3:** Td_5%_ and T_g_ for varying biochar content.

% Carbon	T_d5%_ (°C)	T_g_ (°C)	Char Yield (%)
0	277.877	62.45	7.62
1	273.695	60.65	9.28
5	259.819	54.64	11.56
10	245.012	45.3	14.28
20	236.531	49.12	19.78

**Table 4:** Burn testing results of foams synthesized at the 100 Iso Index.

% Carbon	Burn Rate (mm/s)	After Burn (s)	Cotton Ignited (Y/N)	Observation
0	1.22 ± 0.10	31.22 ± 3.77	Y	Fully burned—Nothing left
1	1.23 ± 0.11	19.97 ± 6.85	Y	Fully burned—Nothing left
5	0.87 ± 0.22	39.06 ± 11.91	N	Fully burned—Char remains
10	0.76 ± 0.21	55.81 ± 14.05	Y	Fully burned—Char remains
20	0.61 ± 0.19	71.77 ± 23.75	Y	Fully burned—Char remains

**Table 5:** Horizontal burn testing results for 10% biochar and decreasing Iso Index.

**Iso Index**	**Burn Rate (mm/s)**	**After Burn (s)**	**Cotton Ignited (Y/N)**	**Observation**
100	0.76	58.8	Y	Fully burned—Residue left
89	1.29	26.1	Y	Fully burned—Residue left
78	0.90	26.1	Y	Fully burned—Residue left
67	0.77	42.4	Y	Fully burned—Residue left
56	1.15	33.0	Y	Fully burned—Residue left
44	0.70	48.0	Y	Fully burned—Residue left
33	0.65	43.3	Y	Fully burned—Residue left

## Data Availability

The original contributions presented in this study are included in the article. Further inquiries can be directed to the corresponding author.
